# Resilient Coping Levels and Psychometric Properties of the Brief Resilient Coping Scale Among Nursing Professionals in Saudi Arabia

**DOI:** 10.3390/healthcare12212181

**Published:** 2024-11-01

**Authors:** Abdulrhman Albougami

**Affiliations:** Department of Nursing Administration, College of Nursing, Majmaah University, Al-Majmaah 11952, Saudi Arabia; a.albougami@mu.edu.sa; Tel.: +966-541-867-498

**Keywords:** brief resilient coping scale, nursing professionals, resilient coping, Saudi Arabia

## Abstract

Objectives: This cross-sectional survey assessed resilient coping levels and their relationship with the sociodemographic characteristics of nursing professionals in Saudi Arabia. Methods: Adult (≥18 years) registered nurses who had been practicing for ≥1 year were included in the study. Resilient coping levels (as assessed via a 4-item Brief Resilience Coping Scale; BRCS) and the data of sociodemographic and other characteristics were collected. Descriptive analysis and ordinal logistic regression were used to analyze the data. Furthermore, the psychometric properties of the BRCS are also reported. Results: Overall, 216 nursing professionals were included. The mean BRCS score was 14.6 (standard deviation = 3.6), with most nursing professionals (62.5%) reporting medium-to-high resilience coping levels. The ordinal logistic regression model demonstrated that nurses with increasing age (*p* = 0.002), best overall health (*p* = 0.001), and in the outpatient department (*p* = 0.049) and intensive care unit (*p* = 0.032) had significantly high resilient coping levels. The internal consistency of the BRCS was good (Cronbach’s alpha = 0.80). The results of the exploratory factor analysis and confirmatory factor analysis clearly indicate a unidimensional solution with one factor. Conclusions: In summary, most nursing professionals in Saudi Arabia showed medium-to-high resilience coping levels. Moreover, this study suggests that the BRCS was found to be a psychometrically reliable and adequate tool for assessing resilience coping levels and provides valuable insights into the relationship between resilient coping levels and the sociodemographic characteristics of nursing professionals in Saudi Arabia.

## 1. Introduction

Nursing professionals are vital to healthcare systems globally, but they experience significant (emotional, physical, and professional) stress that affects their health, patient care, and retention rates [1]. Evidence suggests that work-related stress in the United States alone costs an estimated USD 200–300 million annually, with approximately 90% of employees’ medical issues linked to job stress [2]. This highlights the significant impact of workplace stress on both health and economic resources [3,4,5,6]. Similarly, nursing professionals practicing in Saudi Arabia face significant physical and psychological stress, primarily due to heavy workloads, insufficient resources, and the emotional toll of dealing with death [7,8,9,10].

The concept of resilience emerged in the 1970s, and since then it has been extensively studied in psychology and clinical medicine [11,12]. Resilience refers to an individual’s cognitive and behavioral ability to effectively counter the adverse effects of stress and successfully thrive in the face of adversity or traumatic experiences [13]. Several studies have reported that high resilience levels improve a person’s physical and mental well-being and quality of life [14,15,16,17]. A survey-based study of 2063 individuals found that individuals with high resilience experienced less stress, better psychological responses to stress, and reduced job-related stress in challenging work environments [16]. A recent study found that psychological resilience helps reduce the negative effects of adversities on individuals’ well-being [17]. 

Various scales, including the Dispositional Resilience Scale [18], Resilience Scale [19], and Brief Resilient Coping Scale (BRCS), have been developed to measure resilience [20]. The BRCS, developed in 2004, is a 4-item measure designed to assess adaptive coping tendencies in stressful situations [20]. The BRCS demonstrates strong psychometric properties (i.e., internal consistency [r = 0.76] and test–retest reliability [r = 0.71]), with evidence of convergent validity through its correlations with personal coping resources and psychological well-being [20]. Additionally, the tool has demonstrated strong psychometric properties, including good test-retest reliability over extended periods (4 months to 2 years), indicating that resilient coping is both stable and dynamic [21]. The simplicity and one-dimensional structure of the BRCS makes it a valuable tool for identifying individuals with low resilience who may benefit from targeted interventions across diverse populations [22,23,24,25]. Although the BRCS is one of the most widely used tools for assessing resilience levels, it is an ethnically insensitive instrument. Furthermore, there is a lack of studies examining how age impacts resilience and the lack of invariance research for resilience and other psychological constructs [26,27].

Given the highly stressful profession of nursing professionals, resilience is key to protecting them from personal and occupational stress [28]. Several studies have reported a positive impact of resilient coping on the personal and professional aspects of nursing professionals [29,30,31,32]. However, little is known about the resilience of nursing professionals in Saudi Arabia. Therefore, this study investigated the levels of resilient coping among nursing professionals in Saudi Arabia and examined how these levels relate to their sociodemographic characteristics. Additionally, it evaluated the psychometric properties of the BRCS within this population. This study aims to provide valuable insights into resilience levels within the nursing community and suggests strategies to enhance the healthcare system in Saudi Arabia.

## 2. Methods

### 2.1. Study Design

This cross-sectional survey was conducted between January and May 2023. Adult (18 years or older) registered nursing professionals practicing for at least a year and provided informed consent were eligible for participation in this study. Data were collected at a tertiary care hospital in Riyadh, Saudi Arabia. The protocol and questionnaire used for the survey were reviewed by the Institutional Review Board (IRB) and exempted from IRB approval (IRB number: 21-522E). 

All study participants were informed about the purpose of the survey and assured that the collected information would be kept confidential and used solely for the study. Following approval, data were collected, analyzed, and reported according to the Strengthening the Reporting of Observational Studies in Epidemiology (STROBE) reporting guidelines (Supplementary Materials) (https://www.strobe-statement.org/ accessed on 2 August 2024) [33].

### 2.2. Sociodemographic and Other Characteristics

Sociodemographic characteristics including age, sex, marital status, education, and nationality were collected. Additionally, information on years of clinical experience, work shift, posting ward in the hospital, overall health, and the presence of chronic conditions was recorded.

### 2.3. Assessment of Resilience Coping Levels

Participants’ resilience coping levels were measured using a 4-item BRCS designed to adaptively capture tendencies to cope with stress [20]. Each item on the scale was scored on a 5-point Likert scale from 1 to 5. The total score was summed from the individual item scores and ranges from 4 to 20 (4–13, low resilient coping; 14–16, medium resilient coping; 17–20, high resilient coping) [20].

### 2.4. Statistical Analysis

Sociodemographic characteristics (age, sex, marital status, education level, and nationality) and other characteristics (clinical experience, work shift, posting ward in the hospital, overall health, and the presence of chronic conditions) were analyzed descriptively. Categorical variables are summarized as absolute values and percentages, while continuous variables are presented as means and standard deviations (SDs). Categorical variables were analyzed using the chi-squared test. For continuous measures, a one-way Analysis of Variance (ANOVA) followed by Tukey’s post hoc honestly significant difference test were used to detect differences across the groups. For multivariate analysis, ordinal logistic regression was used to examine the relationship between resilient coping levels and sociodemographic factors with a 95% confidence interval (CI).

The psychometric properties of the BRCS are based on several analyses. As a measure of internal consistency, Cronbach’s alpha was calculated for the BRCS and its four items. Pearson’s correlation (r) was used to measure item correlations. The construct validity of the BRCS was evaluated using an exploratory factor analysis (EFA), a principal component analysis, and varimax rotation. Bartlett’s test of sphericity (expressed as χ^2^) and the Kaiser–Meyer–Olkin (KMO) test were performed. The threshold for Bartlett’s test of sphericity was set to *p* < 0.05, and KMO values were close to 1.0, indicating the usefulness of factor analysis in this study population. The minimum factor-loading criterion was set to 0.50. The commonality of the BRCS (which indicates the amount of variance in each dimension) was also assessed to ensure acceptable explanation levels. Following the EFA, the confirmatory factor analysis (CFA) with maximum likelihood estimation was used to further investigate the construct validity of the BRCS. The goodness of fit for the CFA model was evaluated using various statistical measures, including the χ^2^ test and fit indices such as the Bentler comparative fit index (CFI), Tucker–Lewis Index (TLI), root mean square error of approximation (RMSEA), and standardized root mean square residual (SRMR). For a model to be acceptable, χ^2^ must be non-significant, CFI/TFI values should be around 0.90 (the higher the value, the better the fit), the RMSEA value should be <0.06, and the SRMR value should be <0.08 [34,35,36]. Data analyses were performed using the Statistical Package for the Social Sciences (SPSS, version 26.0. IBM, New York, NY, USA) and R version 4.4.1 with the lavaan package (R Foundation for Statistical Computing, Vienna, Austria).

## 3. Results

### 3.1. Sample Characteristics

A total of 216 nurses (132 in offline mode and 84 in online mode) participated in this study. The mean age of the study participants was 32.0 (SD = 5.3) years, men made up 52.8% of the participants, and 58.8% of participants were married. Participants were predominantly from Saudi Arabia (80.1%), had a bachelor’s degree in nursing (80.6%), and had 6 to 15 years of clinical experience (56.5%). Most nurses worked in the morning shift (60.6%) and were posted in an emergency ward (29.2%), followed by surgical and medical wards. The overall health of most participants (68.5%) was high (i.e., scoring 8–10 on a 10-point rating scale; a lower scale indicated poor health, and a higher score indicated best health), and only 18.5% of the participants reported the presence of a chronic condition.

### 3.2. Resilience Coping Levels

**Figure 1:**
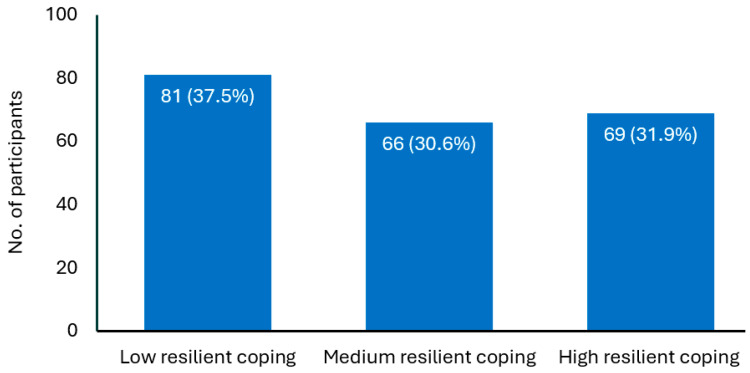
Distribution of study participants based on resilience coping score * (N = 216). Notes: * Measured using the Brief Resilience Coping Scale (4–13 indicates low resilient coping; 14–16 indicates medium resilient coping; 17–20 indicates high resilient coping).

The mean total score of the BRCS among all participants was 14.6 (SD = 3.6), indicating medium coping levels. Most participants reported medium-to-high coping levels (Figure 1).

### 3.3. Relationship Between Resilience Coping Levels and Sociodemographic and Other Variables

When different levels of resilience coping were analyzed in relation to sociodemographic characteristics, a significant association was found with the age of the nursing professionals, i.e., levels of resilience coping increased with the age of the nursing professionals (F = 12.07; *p* = 0.000). Tukey’s post hoc analyses confirmed that nursing professionals with medium and high resilient coping levels had a significantly higher mean age compared to those with low resilience coping. Furthermore, a significant association was found between resilient coping levels and overall health (χ^2^ = 49.34; *p* = 0.000) and the presence/absence of a chronic condition (χ^2^ = 82.22; *p* = 0.000) (Table 1). 

As shown in Table 2, the ordinal logistic regression model demonstrated significant relationships between resilience coping levels and a few characteristics of the nursing professionals. Resilience coping levels showed a positive association with age, indicating that the older the age, the higher the resilience coping levels among nurses (*p* = 0.002). Similarly, nursing professionals posted in outpatient departments (*p* = 0.049), intensive care units (*p* = 0.032), and laboratories (*p* = 0.052) demonstrated significantly higher resilience compared to those posted in other wards. Additionally, coping levels among nursing professionals are linked to their self-assessed overall health, with those rating their health as “best” exhibiting significantly higher coping levels than those in poor (*p* = 0.000) or moderate health (*p* = 0.001) (Table 2).

### 3.4. Psychometric Properties of the BRCS 

The internal consistency (i.e., Cronbach’s alpha) of the BRCS was 0.80, indicating good internal consistency. The scale analysis for Cronbach’s alpha showed item-total correlations between 0.56 and 0.73. Deletion of any of the four items would cause a decrease in the α-value, highlighting the importance of each of the four BRCS items for the scale’s reliability (Table 3).

The EFA performed on the total sample (N = 216) showed significant results (i.e., χ^2^ = 279.78; df = 6; *p* < 0.001), indicating its suitability for factor analysis. The coefficient of the KMO test was 0.74, indicating adequate sampling. Furthermore, the analysis identified a single factor that accounted for 62.33% of the total variance, with factor loadings of 0.75 for BRCS1 and BRCS2, 0.78 for BRCS3, and 0.88 for BRCS4. Subsequently, a CFA was performed on the BRCS items to confirm that resilient coping is the single latent factor influencing the variance and covariance among the four items. The results of the CFAs revealed that the χ^2^ test was significant (18.464; *p* < 0.001), the CFI was 0.994, the TLI was 0.969, the RMSEA was 0.54, and the SRMR was 0.015. Overall, the model fit statistics indicated an acceptable fit to the one-factor model.

## 4. Discussion

To our knowledge, this is the first study to report resilience coping levels of nursing professionals in Saudi Arabia. Overall, the nursing professionals in this study had intermediate BRCS scores, indicating medium resilient coping levels. Most nursing professionals (62.5%) surveyed demonstrated medium-to-high coping levels. Furthermore, sociodemographic correlations of nursing professionals, viz., age, overall health, and posting in certain wards, were significantly associated with the resilience coping levels in this population. Finally, the BRCS is a psychologically reliable and adequate tool to assess resilience coping levels in this population.

There has been an upsurge of interest in understanding resilience capacity and the factors affecting nursing professionals’ coping levels [37,38,39]. Our study found that most nursing professionals in Saudi Arabia exhibited medium to high resilience coping capabilities, which aligns with the resilience levels observed among Brazilian nurses [38]. The analysis of the factors influencing coping levels demonstrated that age was positively associated with levels of resilience (i.e., the older the participants, the greater their resilience coping levels), in alignment with previous studies [38,39]. A cross-sectional study involving 375 nursing workers in Brazil identified age and working time as significant determinants of resilience [38]. Similarly, a UK-based study found that younger and less experienced nurses working in respiratory settings during the COVID-19 pandemic experienced higher levels of anxiety and depression, along with lower resilience [39]. Importantly, the relationship between resilience and age observed in our study contrasts with that reported in other studies [40,41]. The discrepancy observed may be due to differences in the population, study period, and setting in the present study compared to previously published studies [39,40,41]. The overall health of the nursing professionals was another sociodemographic variable that significantly influenced resilience levels, i.e., nursing professionals with the best overall health demonstrated significantly higher coping levels than those with poor or moderate overall health. This finding is similar to a previous study in which students with good perceptions of their health more often showed higher resilience than those with regular or poor perceptions [41]. This is expected, as individuals in the best health state (i.e., physical and psychological) are more engaged and productive and tend to overcome stress more effectively than those in poor health states [42,43]. The socioeconomic status of individuals significantly affects their psychological well-being, with higher status contributing to greater resilience due to better resource access [44]. However, the current study focused on age, health status, and workplace environment in relation to resilience. This emphasizes the need to explore the impact of socioeconomic status on resilience levels in nursing professionals. Further, this study noted that nursing professionals in outpatient departments, intensive care units, and laboratories exhibited higher resilience coping levels compared to those in other wards. This relationship between ward assignment and resilience levels has not been previously documented, indicating a need for further research.

Furthermore, this study investigated the psychometric properties of the BRCS in a nursing population in Saudi Arabia. The internal consistency of the BRCS was good (i.e., Cronbach’s alpha was 0.80), which is consistent with the previous studies that reported Cronbach’s alpha between 0.59 and 0.86 [20,25,27,45,46,47,48]. The scale also presented an adequate inter-total correlation (ranging from 0.56 to 0.73), thus adding to the empirical evidence of the internal consistency of the BRCS in Saudi nursing professionals. The results of the EFA and CFA indicated that the best model for the data was a one-factor solution. Since χ^2^ tests are sensitive to sample size, they tend to reject models in large samples [49]. This should not be considered a criterion to reject the model. The fit indices (i.e., CFI, TLI, RMSEA, and SRMR) for the BRCS model were found to be acceptable, confirming its unidimensional structure, in line with prior studies [25,45,48,50,51]. Because only the BRCS was utilized in the study for assessing resilience coping, an evaluation of construct validity (convergent and discriminant) was not possible. Further research is needed on the construct validity of the BRCS in this population and to assess its cross-cultural applicability for nursing professionals worldwide.

A few caveats in this study merit consideration. The cross-sectional design ruled out causal and longitudinal relationships between resilience coping levels and related indicators. The small sample size from a single tertiary care hospital and convenience sampling add to the bias. Participation in this study was voluntary; therefore, underlying selection bias cannot be ruled out. Moreover, the self-reported nature of the survey may introduce other potential biases (i.e., false reporting and inaccurate recall). Finally, this study reflects the opinions and experiences of nursing professionals in Saudi Arabia; therefore, the findings cannot be generalized to participants from other professions across Saudi Arabia. Further research is needed across various types and regions of healthcare facilities to address the bias from sample selection and improve the applicability of the findings. 

Despite these limitations, the current study adds to our understanding of the association between resilient coping levels and sociodemographic characteristics of nursing professionals in Saudi Arabia. Furthermore, this study used a psychometrically validated and reliable BRCS to assess resilient coping levels, which has been used extensively in several studies. Taken together, our study suggests that there is potential to enhance resilience coping levels among nursing professionals in Saudi Arabia. Implementing targeted interventions focused on emotion regulation, relaxation, psychoeducation, cognitive strategies, and self-compassion may effectively improve their resilience levels [52,53].

## 5. Conclusions

In summary, the nursing professionals in this study exhibited medium-to-high levels of resilient coping, which calls for the development effective strategies that could enhance their coping levels. Moreover, this study suggests that the BRCS is a psychometrically reliable and adequate tool for assessing resilience coping levels and sheds light on the relationship between resilience coping levels and the sociodemographic characteristics of nursing professionals in Saudi Arabia. Further, future studies are warranted to explore specific concerns and devise adequate strategies to improve the resilience coping levels of nursing professionals in Saudi Arabia.

## Figures and Tables

**Table 1:** Relationship between resilience coping levels and sociodemographic and other variables.

Variable	Resilience Coping Levels			Point Estimates	*p*-Value
	Low	Medium	High		
Mean (SD) age [95% CI]	29.9 (5.1) [28.79–31.01]	33.2 (5.0) [31.99–34.41]	33.4 (5.0) [32.22–35.58]	F = 12.07	0.000 ^†^
Sex, n (%)					
Male	40 (35.1%)	37 (32.5%)	37 (32.5%)	χ^2^ = 2.59	0.628
Female	37 (38.5%)	28 (29.2%)	31 (32.3%)		
Prefer not to disclose	4 (66.7%)	1 (16.7%)	1 (16.7%)		
Marital status, n (%)					
Single	28 (42.4%)	18 (27.3%)	20 (30.3%)	χ^2^ = 6.22	0.399
Married	41 (32.3%)	44 (34.6%)	42 (33.1%)		
Divorced	9 (47.4%)	4 (21.1%)	6 (31.6%)		
Widow	3 (75.0%)	0 (0.0%)	1 (25.0%)		
Education, n (%)					
Diploma in nursing	14 (48.3%)	8 (27.6%)	7 (24.1%)	χ^2^ = 2.21	0.697
Graduate in nursing	62 (35.6%)	55 (31.6%)	57 (32.8%)		
Postgraduation in nursing	5 (38.5%)	3 (23.1%)	5 (38.5%)		
Nationality, n (%)					
Saudi	7 (63.6%)	1 (9.1%)	3 (27.3%)	χ^2^ = 7.00	0.320
Filipino	5 (22.7%)	10 (45.5%)	7 (31.8%)		
Indian	66 (38.2%)	52 (30.1%)	55 (31.8%)		
Other	3 (30.0%)	3 (30.0%)	4 (40.0%)		
Clinical experience, n (%)					
≤5 years	33 (47.1%)	17 (24.3%)	20 (28.6%)	χ^2^ = 7.00	0.320
6−14 years	42 (34.4%)	40 (32.8%)	40 (32.8%)		
≥15 years	6 (25.0%)	9 (37.5%)	9 (37.5%)		
Work shift, n (%)					
Morning	49 (37.4%)	43 (32.8%)	39 (29.8%)	χ^2^ = 4.31	0.635
Afternoon	16 (48.5%)	7 (21.2%)	10 (30.3%)		
Evening	8 (33.3%)	8 (33.3%)	8 (33.3%)		
Rotating	8 (28.6%)	8 (28.6%)	12 (42.9%)		
Posting ward, n (%)					
Emergency	19 (46.3%)	12 (29.3%)	10 (24.4%)	χ^2^ = 24.75	0.132
Surgical	26 (53.1%)	14 (28.6%)	9 (18.4%)		
Medical	14 (22.2%)	23 (36.5%)	26 (41.3%)		
OPD	6 (27.3%)	8 (36.4%)	8 (36.4%)		
ICU	4 (22.2%)	6 (33.3%)	8 (44.4%)		
PHC	1 (33.3%)	0 (0.0%)	2 (66.7%)		
Others	2 (66.7%)	0 (0.0%)	1 (33.3%)		
Pediatric	3 (75.0%)	0 (0.0%)	1 (25.0%)		
Obstetrics/Gynecology	3 (37.5%)	2 (25.0%)	3 (37.5%)		
Laboratory	3 (60.0%)	1 (20.0%)	1 (20.0%)		
Overall health *, n (%)					
≤4	20 (80.0%)	4 (16.0%)	1 (4.0%)	χ^2^ = 49.34	0.000
5−7	28 (65.1%)	9 (20.9%)	6 (14.0%)		
8−10	33 (22.3%)	53 (35.8%)	62 (41.9%)		
Presence of a chronic condition **, n (%)					
Yes	21 (25.0%)	55 (65.5%)	8 (9.5%)	χ^2^ = 82.22	0.000
No	60 (45.5%)	11 (8.3%)	61 (46.2%)		

Abbreviations: CI, confidence interval; ICU, intensive care unit; OPD, outpatient department; PHC, primary healthcare. Notes: * Measured on a 0–10 rating scale (score of ≤4 indicates poor health; score between 5 and 7 indicates moderate health; score 8–10 indicates best health). ** Conditions such as hypertension, diabetes, neurological, cardiovascular, and any other disease. ^†^ One-Way Analysis of Variance followed by post hoc Tukey honestly significant difference low vs. medium resilient coping levels (Q = 5.65; *p* = 0.000); low vs. high resilient coping levels (Q = 6.00; *p* = 0.001); medium vs. high resilient coping levels (Q = 0.34; *p* = 0.967).

**Table 2:** Ordinal logistic regression model for the association between the levels of resilience coping and sociodemographic and other characteristics.

Characteristics	Estimate	SE	Walds χ^2^	*p*-Value	95% CI
Resilience coping level					
Low	5.896	2.720	4.699	0.030	0.565 to 11.228
Medium	7.609	2.739	7.719	0.005	2.241 to 12.976
High	0 ^a^	–	–	–	–
Data mode					
Offline	0.422	0.448	0.885	0.347	−0.457 to 1.300
Online	0 ^a^	–	–	–	–
Age	0.166	0.055	9.152	0.002	0.058 to 0.273
Sex					
Male	−0.361	1.148	0.076	0.783	−2.567 to 1.934
Female	0.062	1.126	0.003	0.956	−2.145 to 2.268
Prefer not to disclose	0 ^a^	–	–	–	–
Marital status					
Single	2.169	1.608	1.819	0.177	−0.983 to 5.320
Married	1.891	1.558	1.474	0.225	−1.161 to 4.994
Divorced	2.122	1.653	1.649	0.199	−1.117 to 5.361
Widow	0 ^a^	–	–	–	–
Education					
Diploma in nursing	−1.425	0.826	2.972	0.085	−3.044 to 0.195
Graduate in nursing	−0.793	0.706	1.259	0.262	−2.177 to 0.592
Postgraduation in nursing	0 ^a^	–	–	–	–
Nationality					
Saudi	−0.826	1.074	0.592	0.442	−2.932 to 1.279
Filipino	−0.692	0.934	0.549	0.459	−2.522 to 1.138
Indian	−0.460	0.817	0.317	0.574	−2.061 to 1.141
Other	0 ^a^	–	–	–	–
Clinical experience	−0.072	0.055	2.495	0.114	−0.162 to 0.017
Work shift					
Morning	−0.749	0.466	2.586	0.108	−1.661 to 0.164
Afternoon	−0.840	0.612	1.884	0.170	−2.039 to 0.359
Evening	−0.707	0.646	1.197	0.274	−1.973 to 0.559
Rotating	0 ^a^	–	–	–	–
Posting ward					
Medical	2.159	1.423	2.300	0.129	−0.631 to 4.949
Surgical	2.115	1.384	2.335	0.127	−0.598 to 4.827
Emergency	2.477	1.334	3.449	0.063	−0.137 to 5.091
OPD	2.755	1.398	3.885	0.049	0.016 to 5.494
ICU	2.980	1.394	4.573	0.032	0.249 to 5.712
Laboratory	3.486	1.798	3.761	0.052	0.037 to 7.010
Obstetrics/Gynecology	2.049	1.856	1.219	0.270	−1.589 to 5.687
Pediatric	1.626	1.780	0.834	0.361	−1.863 to 5.116
PHC	1.711	1.420	1.451	0.228	−1.863 to 5.116
Others	0 ^a^	–	–	–	–
Overall health *					
≤4	−2.548	0.613	17.270	0.000	−3.750 to −1.346
5−7	−1.394	0.410	11.582	0.001	−2.197 to −0.591
8−10	0 ^a^	–	–	–	–
Presence of a chronic condition **					
Yes	−0.099	0.415	0.057	0.811	−0.914 to 0.715
No	0 ^a^	–	–	–	–

Abbreviations: CI, confidence interval; ICU, intensive care unit; OPD, outpatient department; PHC, primary healthcare; SE, standard error. Notes: ^a^ This parameter is set to zero because it is redundant. Nagelkerke R^2^ = 0.357; parallel-lines assumption verified (*p* = 0.999). * Measured on a 0–10 rating scale (score of ≤4 indicates poor health; score between 5 and 7 indicates moderate health; score 8–10 indicates best health). ** Conditions such as hypertension, diabetes, neurological, cardiovascular, and any other disease.

**Table 3:** Psychometric properties of Brief Resilient Coping Scale items.

S. No.	BRCS Item	Mean (SD)	Item-Total Correlations	Cronbach’s Alpha If Item Deleted
1	I look for creative ways to alter difficult situations	3.64 (1.06)	0.56	0.77
2	Regardless of what happens to me, I believe I can control my reaction to it	3.65 (1.16)	0.59	0.76
3	I believe I can grow in positive ways by dealing with difficult situations	3.67 (1.17)	0.56	0.77
4	I actively look for ways to replace the losses I encounter in life	3.62 (1.21)	0.73	0.68

Abbreviations: BRCS, Brief Resilient Coping Scale; SD, standard deviation.

## Data Availability

The data presented in this manuscript are available on request from the corresponding author. The data are not publicly available due to privacy reasons.

## Supplementary Materials

The following supporting information can be downloaded at: https://www.mdpi.com/article/10.3390/healthcare12212181/s1, STROBE Statement.
